# Subacute Combined Degeneration Due to Inhalant Use: Not a Laughing Matter

**DOI:** 10.7759/cureus.92459

**Published:** 2025-09-16

**Authors:** Shannon M Lee, Elizabeth Goodman, Jesse Szatkowski

**Affiliations:** 1 Internal Medicine, Harbor UCLA, Torrance, USA

**Keywords:** inhalant use, nitrous oxide use, subacute combined degeneration, substance use disorder (sud), vitamin b12 deficiency

## Abstract

Nitrous oxide, known as "laughing gas," is a substance that can be used to induce analgesia and euphoria. It is easily accessible in certain household products, such as aerosol canisters used for food products. However, chronic use of nitrous oxide can inhibit cobalamin (vitamin B12), causing severe deficiencies that result in psychiatric, hematologic, and neurological toxicities, including subacute combined degeneration of the spinal cord. We present a case of a young adult male who developed progressive, ascending bilateral paresthesias and gait disturbances, ultimately attributed to nitrous oxide use. This case highlights the neurotoxic effects and symptoms associated with recreational nitrous oxide use, as well as the patient's treatment and subsequent clinical recovery.

## Introduction

Nitrous oxide has traditionally been used as an analgesic and anesthetic for surgical procedures. However, beginning in the 1970s, as nitrous oxide became a legal, inexpensive, and easily accessible substance, it began to emerge as a drug of abuse, particularly among young adults, with one study reporting a median age of 27 years old for users [1-3]. It is most often contained within pressurized metal cartridges found in the food industry, such as whipped cream canisters [4]. This led to the use of the term “whippets” to describe nitrous oxide when used recreationally.

Nitrous oxide acts as an irreversible inhibitor of vitamin B12, resulting in severe functional B12 deficiency with neurologic, psychiatric, and hematologic toxicities. Neurologic manifestations occur because vitamin B12 stabilizes the myelin sheath, and its deficiency can lead to demyelination of the dorsal and lateral columns of the spinal cord, resulting in subacute combined degeneration (SCD). SCD of the spinal cord consists of degeneration of the dorsal and lateral white matter of the spinal cord. This often presents as ataxia, lower extremity weakness, and paresthesias [5]. Here, we describe a case of SCD in a patient with a history of recreational nitrous oxide use.

## Case presentation

**Figure 1:**
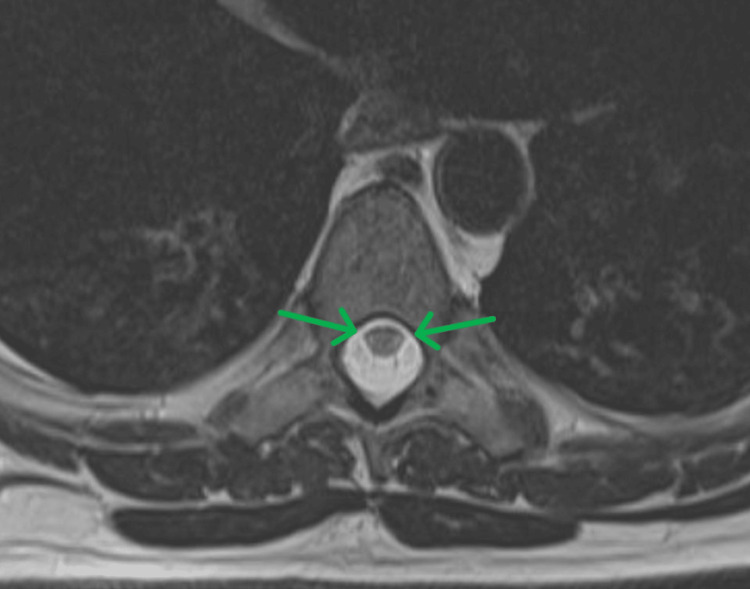
MRI thoracic spine revealing subtle T2 hyperintensity of the lateral columns

A male in their early 30s with a history of seizure disorder, prediabetes, and polysubstance use (including whippets) presented to the emergency department after a ground-level fall. At the time of his hospital presentation, the patient noted several months of progressively worsening ascending bilateral paresthesias, along with gait instability, symmetric lower extremity weakness, and new urinary incontinence. He had previously been evaluated in the primary care clinic for peripheral neuropathy, but he had not disclosed his history of inhalant use. His neurological exam demonstrated involuntary tremor in the left lower leg, decreased lower extremity reflexes, diminished lower extremity muscle strength, absent proprioception and vibratory sense, and a wide-based gait. Laboratory results demonstrated a normal complete blood count (hemoglobin, hematocrit, and platelet count) and normal folate. Vitamin B12 was reduced, while methylmalonic acid was markedly elevated. The full laboratory values and reference ranges are shown in Table 1. MRI showed mild cervical degenerative changes along with subtle T2 hyperintensity of the lateral columns at T7-T8, consistent with the diagnosis of SCD (Figure 1). The patient was treated with vitamin B12 injections and discharged to an acute rehabilitation unit (ARU). Cessation from nitrous oxide was recommended. At his six-month follow-up, neurological function had significantly improved, and his vitamin B12 level had improved to 156 pg/mL. Testing for intrinsic factor antibodies and tissue transglutaminase antibodies was negative.

**Table 1:** Laboratory values at initial presentation

Lab Test	Patient Lab Values	Reference Range
Hemoglobin	13.9 g/dL	13.5-16.5 g/dL
Hematocrit	41.9%	40-49%
Platelet Count	236 K/cumm	160-360 K/cumm
Vitamin B12	74 pg/mL	190-150 pg/mL
Folate	31 ng/mL	>5.9 ng/mL
Methylmalonic Acid	17,160 nmol/L	87-318 nmol/L

## Discussion

Like many inhalants, the popularity of nitrous oxide is thought to be driven by its low cost and easy accessibility [5]. Nitrous oxide works within seconds of inhalation to induce analgesia and euphoria, as well as hallucinogenic effects. It is rapidly cleared and thus does not cause an unpleasant “hangover” effect. Its association with euphoria is likely due to a combination of its interactions with opioid pathways, along with antagonism of the N-methyl-D-aspartate (NMDA) receptor, leading to dopamine disinhibition. Given how quick the effects are, nitrous oxide abusers sometimes inhale repeatedly without awareness of the long-term repercussions [6].

Nitrous oxide-associated vitamin B12 deficiency has been described to occur as soon as one month after the onset of heavy use [7]. There are a limited number of documented cases of nitrous oxide causing SCD [5]. In addition to SCD, chronic nitrous oxide use has been associated with peripheral neuropathy, megaloblastic anemia, myelosuppression, memory loss, and psychosis. For patients with concerns for chronic nitrous oxide abuse, work-up should include vitamin B12, methylmalonic acid (MMA), and homocysteine levels [4,8]. However, it is important to recognize that vitamin B12 levels are sometimes normal, as the deficiency is considered functional due to its interference with vitamin B12’s function as a coenzyme. Nitrous oxide-associated SCD is treated with vitamin B12, typically by intramuscular injection of 1,000 µg every other day for a week, weekly for four to eight weeks, and monthly until replete [6,9]. When treated early with vitamin B12 injections, neurologic toxicities can often be reversed, as in our patient. However, delays in treatment can be associated with permanent disability [10].

Nitrous oxide is one of several addictive inhalant drugs increasingly used among adolescents and young adults. Inhalant drug use is a rapidly expanding epidemic with three-quarters of a million new users in the United States annually, 58% of whom initiated use in the ninth grade or below. Inhalant use disorders are underdiagnosed, likely due to a lack of clinical suspicion combined with the absence of definitive diagnosis through laboratory testing [11]. In addition, it is easily disguised behind commonly used items, such as hair spray, glue, lighter fluid, nail polish remover, and whipped cream [11,12]. Patients also may not readily identify that their symptoms are a sequelae of their nitrous use.

Currently, there are no approved medication-assisted therapies for nitrous oxide abuse. There are only a few studies that have addressed inhalant use disorders or their treatment, leading it to be referred to as “the forgotten epidemic” [11]. Awareness of inhalants as a drug of abuse by clinicians is vital to early diagnosis, prevention, and treatment of their complications, particularly as patients may be reluctant to report use, likely due to the stigma associated with substance use disorders. Several validated questionnaires, such as the Alcohol, Smoking and Substance Involvement Screening Test (ASSIST) questionnaire, do include specific questions regarding inhalant use, which may provide an opportunity to identify patients who use nitrous oxide [13].

## Conclusions

Overall, our patient had a classic presentation of nitrous oxide use; however, a thorough social history is essential to uncover inhalant use disorder. Validated screening questionnaires are important tools for clinicians to ensure all potential etiologies are considered. Fortunately, our patient sought treatment, abstained from further nitrous oxide use, and completed physical therapy at an acute rehabilitation unit. He subsequently showed significant improvement in his functional status. It is important to consider nitrous oxide use in patients who present with neurologic symptoms consistent with SCD.
